# Focal points of preanesthesia evaluations for electroconvulsive therapy in patients with depression: a retrospective analysis of clinical characteristics in nonremission

**DOI:** 10.1186/s12871-022-01686-6

**Published:** 2022-05-26

**Authors:** Lei Zou, Xiao Li, Qibin Chen, Feng Lv, Su Min

**Affiliations:** 1 Department of Anesthesiology, The First Affiliated Hospital of Chongqing Medical University, Chongqing, 400016 China; 2 Departments of Psychiatry, the First Affiliated Hospital of Chongqing Medical University, Chongqing, China

**Keywords:** ECT, Retrospective analysis, Nonremission, Depression

## Abstract

**Background:**

This study explored the patient clinical characteristics that may affect electroconvulsive therapy (ECT) efficacy to enable improved focus during evaluations and preparation for ECT.

**Methods:**

Patients were enrolled for ECT at the Department of Psychiatry and Anesthesiology of the First Affiliated Hospital of Chongqing Medical University from December 2017 to January 2019. The primary outcome in our study was defined as the development of nonremission. A multivariate logistic analysis was performed to identify the risk factors for nonremission.

**Results:**

In total, 874 depressed patients were included in the study. After the ECT treatment, 255 cases (29.2%) exhibited nonremission. A multivariate logistic regression analysis of the variables was performed, and the results showed that atherosclerosis (OR 8.072, 95% CI 2.442 to 16.675; *P* = 0.001), COPD (OR 2.919, 95% CI 1.240 to 6.871; *P* = 0.014), diabetes (OR 2.202, 95% CI 1.115 to 4.348; *P* = 0.023) and smoking (OR 1.519, 95% CI 1.015 to 2.273; *P* = 0.042) were independent risk factors for nonremission.

**Conclusion:**

In the retrospective analysis, we found that atherosclerosis, diabetes, COPD and smoking may be high-risk factors for nonremission.

**Supplementary Information:**

The online version contains supplementary material available at 10.1186/s12871-022-01686-6.

## Introduction

Depression is a mood disorder characterized by persistent feelings of loss of interest along with a cluster of clinical symptoms. The World Health Organization projects that worldwide, depression will be the leading cause of disease burden by 2030, and depression is a significant public health concern affecting 350 million people worldwide [1, 2]. Despite the development of newer brain stimulation techniques and novel pharmacological agents, no treatment has approached the efficacy of electroconvulsive therapy (ECT) for depression, especially among patients whose medical or psychiatric condition requires a rapid and/or definite response [3], and researchers have attempted to identify other methods to improve the efficacy of ECT.

Anesthesia is an indispensable part of ECT treatment as it not only eliminates the patients’ fear of the procedure, thereby reducing the stress response, but also reduces the incidence of adverse events, such as fracture, tooth injury, and cardiovascular and cerebrovascular accidents [4–6]. Furthermore, the rational use of anesthetics, such as propofol and ketamine, can improve the efficacy of ECT [7–9] and may confer at least a short-term advantage in terms of improving depressive symptoms at the early stages of ECT [10]. A series of studies conducted by our research group also supported these views [11, 12].

We studied the effects of ketamine in different age groups and confirmed its validity and safety. In addition, by referring to the literature, we found that ECT was particularly effective in elderly individuals with depression [13–15]. However, there are differing views. Some studies have suggested that the response and remission rates to pharmacotherapy and ECT do not sufficiently differ between old-age and middle-age depression patients to be clinically significant [16] or that their effectiveness is independent of age [17]. In addition, elderly individuals may have more complications, such as hypertension, diabetes, atherosclerosis, and hyperlipidemia, which may aggravate the depressive symptoms of these patients [18–20].

Combined with our research and current reports, we believe that the patient’s age, medical complications, adjuvant anesthesia and other factors (i.e., gender, education level, marital status, living habits, and smoking or drinking history) affect the efficacy of ECT and may explain why depression is not alleviated after treatment with ECT. However, a comprehensive analysis of these factors is lacking. The purpose of this study was to explore the risk factors associated with the efficacy of ECT to identify factors that may be addressed during preoperative anesthetic evaluations.

## Methods

### Study patients

This study retrospectively enrolled patients who underwent ECT at the Department of Psychiatry and Anesthesiology of the First Affiliated Hospital of Chongqing Medical University from December 2017 to January 2019. The study protocol was approved by the ethics committee of the hospital, and all aspects of the study complied with the Declaration of Helsinki. There was no need to obtain informed consent from the patients since this was a retrospective study, and all data were collected and analyzed anonymously. The inclusion criteria were complete medical records, and subjects diagnosed with depression based on the DSM-IV-TR [21] were included. Patients diagnosed with other psychiatric diseases were excluded. Furthermore, outpatients were excluded due to incomplete information.

### Data sources

We collected the patients’ clinical data using the electronic medical record system; all data were prospectively input by our clinicians. The basic information of the patients, i.e., sex, body mass index (BMI), smoking history, drinking history, duration of disease, marital status, and recurrent depression, was collected. The education level was divided into illiterate, nine-year compulsory education and high school, and above. The smoking status was classified as nonsmoking and smoking. Additionally, the participants were divided into a nondrinking group and a drinking group. Marital status was classified as unmarried, married, divorced or widowed. According to their age, the participants were classified as minor (< 18 years), youth (18–44 years), middle age (45–59 years) and old age (≥60 years). The physical examination included measurements of height and weight. We measured height and weight to the nearest 1 cm and 0.1 kg, respectively. The BMI was calculated as the weight in kilograms divided by the height in meters squared. Individuals with BMIs≥24 kg/m^2^ were considered overweight, and those with BMIs≥28 kg/m^2^ were considered obese. Comorbidities included diabetes, hypertension, atherosclerosis, hyperlipidemia, chronic obstructive pulmonary disease (COPD), and hypothyroidism.

Depression was ascertained on the basis of the Diagnostic and Statistical Manual of Mental Disorders, Fourth Edition. Depressive symptoms were assessed using the 24-item Hamilton Depression Rating Scale (HAMD-24) at baseline and after the end of the ECT session.

### Primary outcome

The primary outcome in our study was the development of nonremission, which was defined as a HAMD-24 score > 10 after the last two consecutive ECT sessions.

### Statistical analysis

A Shapiro–Wilk test was used to determine whether the continuous data were normally distributed. All continuous data are presented as the median (interquartile range, IQR) and were compared using a Mann–Whitney U test. The categorical data are presented as frequencies and percentages, and the comparisons were achieved by a Pearson χ^2^ test or Fisher’s exact test.

Multivariate logistic analyses were performed to identify the risk factors for the primary endpoint of nonremission. The model was fitted with adjustment for patient-level candidate variables, including age, sex, marital status, education, BMI, smoking, alcohol use, diabetes, hypertension, atherosclerosis, hyperlipidemia, COPD, hypothyroidism, history of anesthesia, family history of depression, first-onset depression, duration of depression and number of electroconvulsive therapies. Multicollinearity among the covariates was examined using the variance inflation factor with a reference value of 10, and no collinearity was observed (Supplemental Table). The results are presented as the odds ratios (ORs) and 95% confidence intervals (CIs).

*P* values< 0.05 were considered statistically significant. The statistical analyses were performed using IBM SPSS Statistics 26.0 (IBM Corporation, Armonk, NY) and R software 4.2.0 (R Project for Statistical Computing, Vienna, Austria, 2021).

## Results

**Fig. 1:**
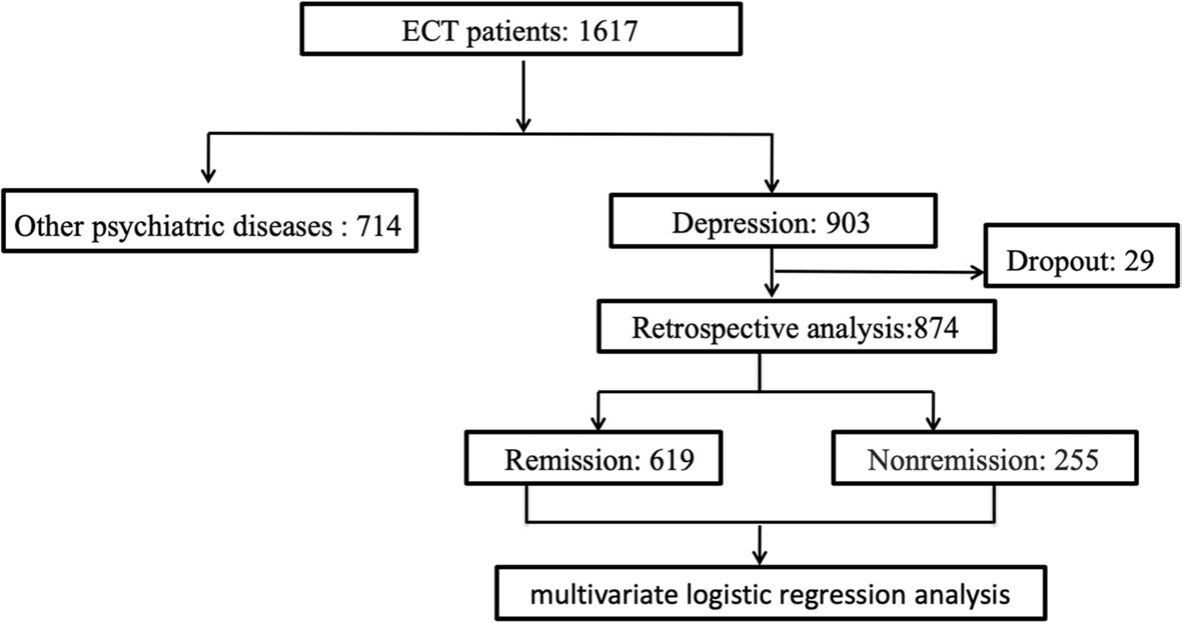
Clinical research flow chart

During this period, 1617 patients underwent ECT; of these patients, 903 were diagnosed with depression. Because not all information could be obtained for outpatients, 874 inpatients were ultimately included. According to the diagnostic criteria, 619 patients achieved remission, with a remission rate of 70.8%. In total, 255 people did not reach the remission standard, accounting for 29.2% of the patients (Fig. 1).

The patients’ clinical characteristics are shown in Table 1. There were no significant differences between the patients with remission and those without remission in sex (*P* = 0.774), education (*P* = 0.304), alcohol use (*P* = 0.199) and family history of depression (*P* = 0.687). Compared with the patients with remission, those without remission were relatively older (*P* = 0.003), had a higher BMI (*P* = 0.018), had fewer marriages (*P* = 0.018) and were smokers (*P* = 0.014). Furthermore, the patients who developed nonremission were characterized by more comorbidities at baseline, including diabetes (*P* < 0.001), hypertension (*P* = 0.002), atherosclerosis (*P* < 0.001), COPD (*P* = 0.005), hyperlipidemia (*P* < 0.001), and hypothyroidism (*P* = 0.038). Additionally, the patients who had remission had a higher frequency of anesthesia history (*P* = 0.014) and a lower rate of first-onset depression (*P* = 0.004) than those who had no remission.

**Table 1:** Demographic characteristics of the remission group and the nonremission group

	Remission (*n* = 619)	Nonremission (*n* = 255)	χ2	P
Age (year)			14.093	0.003
Minor	111 (17.9)	29 (11.4)		
Youth	289 (46.7)	109 (42.7)		
Middle age	145 (23.4)	66 (25.9)		
Old age	74 (12.0)	51 (20.0)		
Gender			0.083	0.774
Male	217 (35.1)	92 (36.1)		
Female	402 (64.9)	163 (63.9)		
Marital status			10.318	0.018
Unmarried	257 (41.5)	79 (31.0)		
Married	321 (51.9)	150 (58.8)		
Divorced	28 (4.5)	19 (7.5)		
Widowed	13 (2.1)	7 (2.7)		
Education			2.379	0.304
Illiterate	29 (4.7)	16 (6.3)		
9-years compulsory education	321 (51.9)	119 (46.7)		
High school or above	269 (43.5)	120 (47.1)		
BMI			8.030	0.018
< 18.5	20 (3.2)	5 (2.0)		
18.5–23.9	565 (91.3)	223 (87.5)		
≥24	34 (5.5)	27 (10.6)		
Smoking			6.100	0.014
Yes	89 (14.4)	54 (21.2)		
No	530 (85.6)	201 (78.8)		
Alcohol use			1.647	0.199
Yes	88 (14.2)	45 (17.6)		
No	531 (85.8)	210 (82.4)		
Diabetes			13.863	< 0.001
Yes	22 (3.6)	25 (9.8)		
No	597 (96.4)	230 (90.2)		
Hypertension			9.169	0.002
Yes	43 (6.9)	34 (13.3)		
No	576 (93.1)	221 (86.7)		
Atherosclerosis			25.586	< 0.001
Yes	4 (0.6)	16 (6.3)		
No	615 (99.4)	239 (93.7)		
Hyperlipidemia			13.416	< 0.001
Yes	27 (4.4)	28 (11.0)		
No	592 (95.6)	227 (89.0)		
COPD			7.893	0.005
Yes	12 (1.9)	14 (5.5)		
No	607 (98.1)	241 (94.5)		
Hypothyroidism			4.301	0.038
Yes	27 (4.4)	20 (7.8)		
No	592 (95.6)	235 (92.2)		
History of anesthesia			5.979	0.014
Yes	156 (25.2)	85 (33.3)		
No	463 (74.8)	170 (66.7)		
Family history of depression			0.163	0.687
Yes	94 (15.2)	36 (14.1)		
No	525 (84.8)	219 (85.9)		
First-onset depression			8.231	0.004
Yes	238 (38.4)	72 (28.2)		
No	381 (61.6)	183 (71.8)		
Duration of depression, median (IQR), months	15 (5,48)	36 (8,75)	24.099^a^	< 0.001
Number of ECT, median (IQR), times	10 (8,12)	9 (7,12)	28.031^a^	< 0.001

*BMI* Body mass index, *COPD* Chronic obstructive pulmonary disease, *ECT* Electroconvulsive therapy, *IQR* Interquartile range

^a^ represents the Z value of the continuous data

**Fig. 2:**
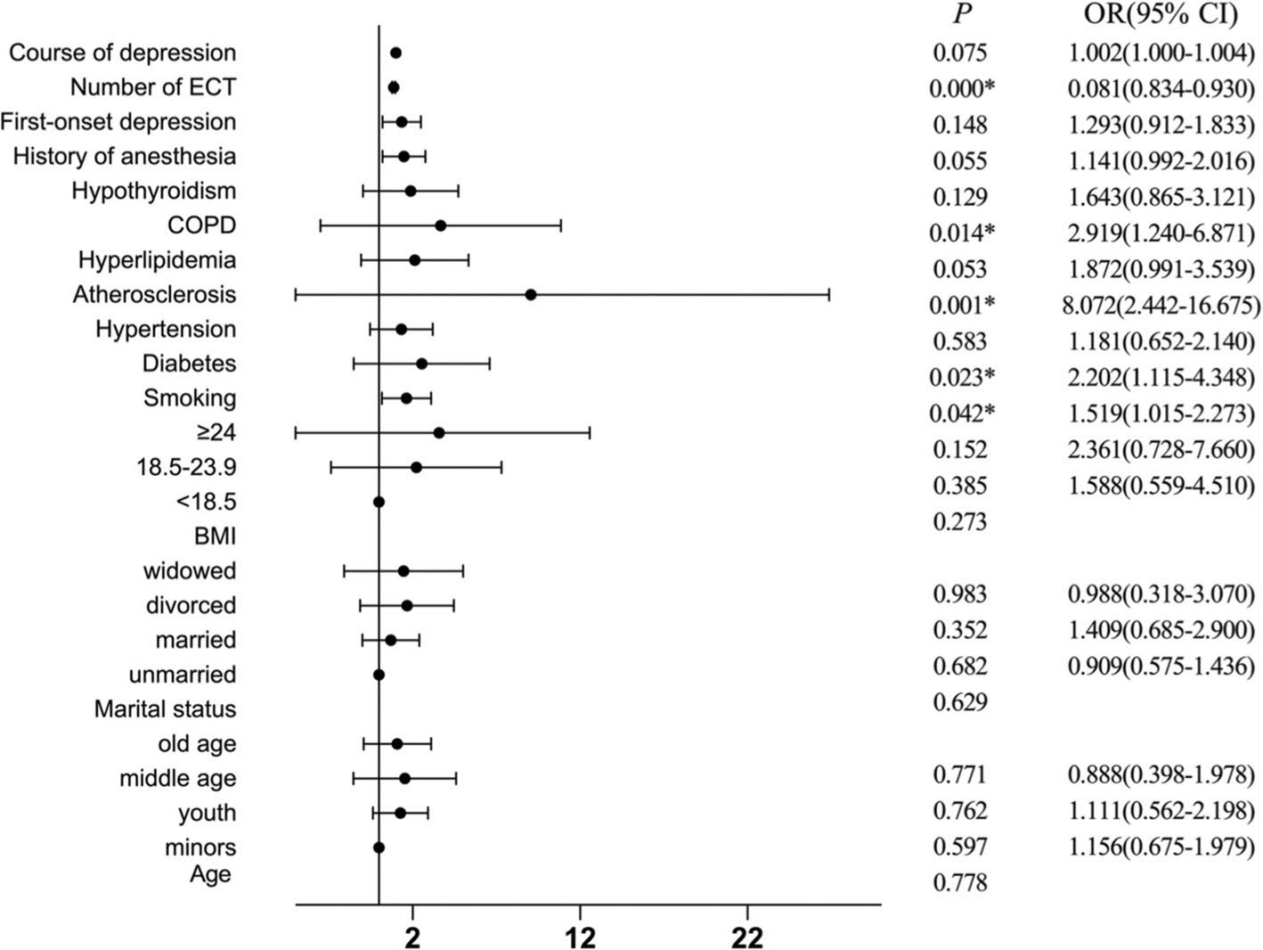
Multivariate analyses of the risk factors for nonremission

The multivariate logistic regression analysis revealed that atherosclerosis (OR 8.072, 95% CI 2.442–16.675, *P* = 0.001), COPD (OR 2.919, 95% CI 1.240–6.871, *P* = 0.104), diabetes (OR 2.202, 95% CI 1.115–4.348, *P* = 0.023) and smoking (OR 1.519, 95% CI 1.015–2.273, *P* = 0.042) were independent risk factors for nonremission. (Table 2, Fig. 2).

**Table 2:** Multivariate analysis of the risk factors for nonremission

Variates	B	OR (95% CI)	P
Age			
Minors		1 (reference)	0.778
Youth	0.145	1.156 (0.675–1.979)	0.597
Middle age	0.105	1.111 (0.562–2.198)	0.762
Old age	−0.119	0.888 (0.398–1.978)	0.771
Marital status			
Unmarried		1 (reference)	0.629
Married	−0.096	0.909 (0.575–1.436)	0.682
Divorced	0.343	1.409 (0.685–2.900)	0.352
Widowed	−0.012	0.988 (0.318–3.070)	0.983
BMI			
< 18.5		1 (reference)	0.273
18.5–23.9	0.462	1.588 (0.559–4.510)	0.385
≥24	0.859	2.361 (0.728–7.660)	0.152
Smoking			
No		1 (reference)	
Yes	0.418	1.519 (1.015–2.273)	0.042
Diabetes			
No		1 (reference)	
Yes	0.789	2.202 (1.115–4.348)	0.023
Hypertension			
No		1 (reference)	
Yes	0.167	1.181 (0.652–2.140)	0.583
Atherosclerosis			
No		1 (reference)	
Yes	2.088	8.072 (2.442–16.675)	0.001
Hyperlipidemia			
No		1 (reference)	
Yes	0.627	1.872 (0.991–3.539)	0.053
COPD			
No			
Yes	1.071	2.919 (1.240–6.871)	0.014
Hypothyroidism			
No		1 (reference)	
Yes	0.496	1.643 (0.865–3.121)	0.129
History of anesthesia			
No		1 (reference)	
Yes	0.346	1.414 (0.992–2.016)	0.055
First-onset depression			
No		1 (reference)	
Yes	0.257	1.293 (0.912–1.833)	0.148
Duration of depression (1 month increase)	0.002	1.002 (1.000–1.004)	0.075
Number of ECT (1 time increase)	−0.127	0.881 (0.834–0.930)	< 0.001

*OR* Odds ratio, *CI* Confidence interval, *BMI* Body mass index, *COPD* Chronic obstructive pulmonary disease, *ECT* Electroconvulsive therapy

## Discussion

Currently, research investigating how to improve the efficacy of ECT is carried out mainly by psychiatrists exploring aspects, such as the electroshock mode, electric quantity and stimulation site [22], while anesthesiologists are mainly concerned with different anesthetic compounds or administration modes [23, 24]. By analyzing the clinical characteristics of nonremission patients, this study intended to improve the evaluation and regulation of patients before anesthesia to further improve the efficacy of ECT. We found that atherosclerosis, diabetes, COPD and smoking may be high-risk factors for nonremission ECT. To date, there is no relevant research, but it is certain that these factors can affect the symptoms of depression.

There is an interactive relationship between depression and atherosclerosis. Studies have shown that depression increases the risk of atherosclerosis. First, from the perspective of pathophysiology, depression is accompanied by immune dysregulation [25], and in this context, the levels of various peripheral inflammatory biomarkers are increased [26]. Furthermore, there are alterations in the nitric oxide (NO) system [27]. Endothelium-derived NO, through its vasodilator properties, participates in the modulation of vascular tone [28]. These factors promote atherosclerosis. In addition, the behavioral effects of depressive symptoms, such as sleep disorders, sedentary behavior and obesity, smoking and alcohol consumption, are considered another source of subclinical atherosclerosis. Sleep disorders lead to impaired vascular endothelial function [29], sedentary behavior causes intimal thickening of arteries [30], and smoking and obesity lead to the formation of atherosclerotic plaques [31], all of which contribute to an increased atherosclerotic burden. Depression and atherosclerosis have a common pathophysiological basis [32, 33], and it has been confirmed that intracranial atherosclerosis aggravates the symptoms of depression [34]. It has been reported that depression patients with atherosclerosis have a poor response to drug treatment [35]. However, ECT treatment has not been reported. Our study found that atherosclerosis is the most important reason for nonremission after ECT, showed that atherosclerosis may have an impact on depression in another way, and indicated that atherosclerosis can influence the treatment efficacy. Some studies have shown that the treatment of atherosclerosis can reduce depression [36]. Therefore, we should treat atherosclerosis when evaluating such patients as this may improve the efficacy of ECT.

Evidence also suggests that a bidirectional relationship exists between diabetes and depression [37, 38]. Numerous studies have confirmed that the duration of depression in patients with diabetes is more severe and that depression episode relapses are more frequent. From a pathophysiological perspective, depression is highly consistent with diabetic complications. These complications include macrovascular complications (such as coronary artery disease), microvascular complications (such as diabetic retinopathy, neuropathy, nephropathy or end-stage renal disease) and bidirectional complications (depression may increase the risk of diabetic complications). Furthermore, these complications affect the occurrence and development of depression [20, 39]. Our research also shows that diabetes is a high-risk factor affecting the efficacy of ECT, which may be related to the aggravation of depressive symptoms by diabetic complications. The treatment of diabetes can reduce the symptoms of depression [40], which may help improve the efficacy of ECT. However, prospective studies supporting this conclusion are lacking.

Our study also found that COPD and smoking are key indicators affecting the efficacy of ECT. These two factors differ from the previous two high-risk factors. Although these factors do not directly cause vascular disease, they affect the development of depression in other ways. COPD causes hypoxia by destroying the lung capillary bed and causing poor airway ventilation, leading to a decrease in neurotransmitter serotonin activity and eventually depression [41, 42]. Hypoxia may also cause the global suppression of cerebral metabolism (energy production), leading to depression [43, 44]. A series of symptoms of COPD also causes depression, and dyspnea, as the core symptom of COPD, may play an important role in the causal relationship between COPD and depression [45, 46]. These patients are less active, have a lower quality of life and have a worse mood. Smoking has an impact on vascular disease and lung disease, aggravating these diseases [47, 48]. Smoking may also be an independent risk factor for depression [49, 50]. This may be the reason why smoking affects the efficacy of ECT.

The treatment of these risk factors (i.e., atherosclerosis and diabetes) can improve the depressive symptoms of patients, but the correlation between these factors and ECT has not been reported. We are conducting prospective studies to assess the impact on ECT by intervening in related risk factors. In addition, psychiatrists can improve the efficacy of ECT by changing the stimulation power, using different stimulation sites, changing the stimulation mode, etc. [22, 51]. Therefore, we hope that when anesthesiologists evaluate patients, they will pay attention to and address these risk factors to further improve the efficacy of ECT.

### Limitations

There were several limitations in this study. First, although these risk factors certainly have an effect on depression, we were unable to confirm their direct relationship with the risk of nonremission. Second, we could not rule out interactions among these factors. Third, currently, prospective studies confirming these findings are lacking. Finally, the study included factors with which anesthesiologists can intervene, and we did not consider different degrees of depression and ECT techniques as risk factors. Therefore, in the next phase of the study, we aim to use the experience of enhanced postoperative recovery (ERAS) to treat depression patients who have these risk factors during anesthesia evaluation. We are also working with psychiatrists to improve the research.

## Supplementary Information

**Additional file 1.** Supplemental Table. The variance inflation factor values of variates included in the multivariate analysis.

## Data Availability

The datasets generated during the current study are not publicly available due to patient privacy concerns but are available from the corresponding author upon reasonable request.
