# Supplementary material for: Focal points of preanesthesia evaluations for electroconvulsive therapy in patients with depression: a retrospective analysis of clinical characteristics in nonremission
Source: BMC Anesthesiol. 2022 May 26;22:163. doi: 10.1186/s12871-022-01686-6 (PMC9134596; doi:10.1186/s12871-022-01686-6)
Supplement: Supplementary file 1 — Additional file 1. Supplemental Table. The variance inflation factor values of variates included in the multivariate analysis. [file 12871_2022_1686_MOESM1_ESM.docx]

| Supplemental Table. The variance inflation factor values of variates included in the multivariate analysis. | |
| --- | --- |
| Variates | Variance inflation factor |
| Age | 1.94 |
| Sex | 1.24 |
| Marital status | 1.56 |
| Education | 1.06 |
| BMI | 1.05 |
| Smoking | 1.48 |
| Alcohol use | 1.42 |
| Diabetes | 1.15 |
| Hypertension | 1.23 |
| Atherosclerosis | 1.13 |
| Hyperlipidemia | 1.14 |
| COPD | 1.03 |
| Hypothyroidism | 1.02 |
| History of anesthesia | 1.11 |
| Family history of depression | 1.12 |
| First-onset depression | 1.11 |
| Duration of depression | 1.59 |
| Number of electroconvulsive therapy | 1.35 |
| BMI, body mass index; COPD, chronic obstructive pulmonary disease. | |
